# ChREBP promotes the differentiation of leukemia-initiating cells to inhibit leukemogenesis through the TXNIP/RUNX1 pathways

**DOI:** 10.18632/oncotarget.9520

**Published:** 2016-05-20

**Authors:** Hongxiang Zeng, Hao Gu, Chiqi Chen, Minle Li, Fangzhen Xia, Li Xie, Xiaoye Liu, Feifei Zhang, Xuemei Tong, Jiangbo Wang, Zhuo Yu, Junke Zheng

**Affiliations:** ^1^ Hongqiao International Institute of Medicine, Shanghai Tongren Hospital, Key Laboratory of Cell Differentiation and Apoptosis of Chinese Ministry of Education, Shanghai Jiao Tong University School of Medicine, Shanghai, China; ^2^ Institute of Health Sciences, Shanghai Institutes for Biological Sciences, Chinese Academy of Sciences, University of Chinese Academy of Sciences, Shanghai, China; ^3^ Department of Biochemistry and Molecular Cell Biology, Shanghai Key Laboratory of Tumor Microenvironment and Inflammation, Shanghai Jiao Tong University School of Medicine, Shanghai, China; ^4^ Department of Hematology, Xinhua Hospital, Shanghai Jiao Tong University School of Medicine, Shanghai, China

**Keywords:** ChREBP, TXNIP, leukemia initiating cells, differentiation, metabolism

## Abstract

Targeting leukemia-initiating cells (LICs) is the key to eradicating leukemia and preventing its relapse. Recent studies have indicated that metabolic regulation may play a critical role in the maintenance of stemness in LICs, although the detailed mechanisms are poorly understood. Herein, we provide intriguing evidence showing that a glucose-responsive transcription factor, carbohydrate responsive element binding protein (ChREBP), served as a tumor suppressor rather than an oncogene, as previously described, to inhibit the development of acute myeloid leukemia by promoting the differentiation of LICs. Using an MLL-AF9-induced murine leukemia model, we demonstrated that the deletion of ChREBP resulted in the blockage of the differentiation of LICs and significantly reduced survival in ChREBP-null leukemic mice. However, ChREBP was not required for the normal repopulation abilities of hematopoietic stem cells. ChREBP promoted leukemia cell differentiation through the direct inhibition of RUNX1 or the transactivation of TXNIP to downregulate the RUNX1 level and ROS generation. Moreover, knockdown of ChREBP in human leukemia THP1 cells led to markedly enhanced proliferation and decreased differentiation upon PMA treatment. Collectively, we unraveled an unexpected role of ChREBP in leukemogenesis, which may provide valuable clues for developing novel metabolic strategies for leukemia treatment.

## INTRODUCTION

Acute myeloid leukemia (AML) is mainly caused by the accumulation of a variety of genetic mutations, such as chromosome translocations, deletions and point mutations. Mixed-lineage leukemia (MLL) rearrangements commonly occur in many subtypes of AML and infant acute lymphoblastic leukemia [1]. Recent studies have indicated that leukemia-initiating cells (LICs) can continuously self-renew and further differentiate to leukemia blasts or more mature leukemia cells to sustain leukemia occurrence and development. LICs have also been reported to be resistant to chemotherapy and radiotherapy and to be responsible for the relapse of leukemia [2, 3]. Several lines of evidence suggest that metabolic changes may be tightly associated with the stemness (self-renewal and differentiation) of LICs as well as that of other types of cancer stem cells [4–7]. Understanding the metabolic regulations involved in the stemness of LICs is critical for the identification of specific targets to effectively eliminate different types of leukemia.

In the past decades, multiple oncogenic pathways have been found to be involved in metabolic alterations during cancer development. For example, PI3K/AKT/mTOR signaling is one of the most commonly altered pathways in human cancers, including leukemia and lymphoma. Studies have shown that the dysregulation of many genes that are involved in glycolysis or oxidative phosphorylation, such as glucose transport protein 1 (GLUT1), glucose-6-phosphate dehydrogenase (G6PD) and isocitrate dehydrogenase 1/2 (IDH1/2), leads to a high risk of the occurrence of leukemia or other tumors [8–11]. Although it is well known that cancer cells tend to use aerobic glycolysis even under normoxic conditions (Warburg's effect) [12], studies by Eleni D. Lagadinou and colleagues have illustrated that, instead of glycolysis, LICs utilize oxidative phosphorylation as the main energy source through the upregulation of BCL-2 [13]. More effort is required to unravel the relationship between LIC stemness and the metabolism of different nutrients.

Carbohydrate response element binding protein (ChREBP) is a large transcription factor containing approximately 850 amino acids that is highly conserved among species [14]. ChREBP contains several critical functional domains, such as a DNA-binding motif of the bHLH/ZIP type and proline-rich regions. ChREBP has been reported to be a key transcription factor that regulates both glycolysis and lipogenesis in hepatocytes [15, 16]. A high glucose level leads to the dephosphorylation of ChREBP, followed by its translocation into the nucleus and binding to the promoters of target genes related to glycolysis and lipogenesis, including L-PK, ACC, FAS, ACC1, SCD1 and TXNIP [17]. Evidence has shown that ChREBP promotes glycolysis and proliferation in both normal cells and solid tumors. For example, ChREBP is critical for the glucose-stimulated proliferation of pancreatic β-cells [18]. Knockdown of ChREBP caused a remarkable decrease in glycolysis, lipogenesis and the synthesis of nucleic acids but an increase in oxidative phosphorylation and the ROS level to inhibit the growth of colorectal cancer cell lines [19]. It is well known that certain ROS levels may be critical for many physiological activities. However, a high level of ROS may enhance the differentiation or reduce the proliferation of cancer cells [20, 21]. How ChREBP controls the ROS levels and the metabolic functions of ChREBP in LICs remain largely unknown.

Herein, we demonstrate that ChREBP is highly upregulated in mouse AML cells (as well as LICs) and acts as a tumor suppressor, rather than as an oncogene as previously reported, to inhibit the differentiation of LICs from a MLL-AF9-induced murine AML model. ChREBP deletion led to notably accelerated leukemia progression. ChREBP collaborated with TXNIP and RUNX1 to promote LIC differentiation. This study unravels a differential role of ChREBP in leukemia development compared to that in other solid tumors, which may open a new avenue for the treatment of leukemia or other types of cancers.

## RESULTS

### ChREBP serves as a tumor suppressor to inhibit leukemogenesis in a murine AML model

**Figure 1:**
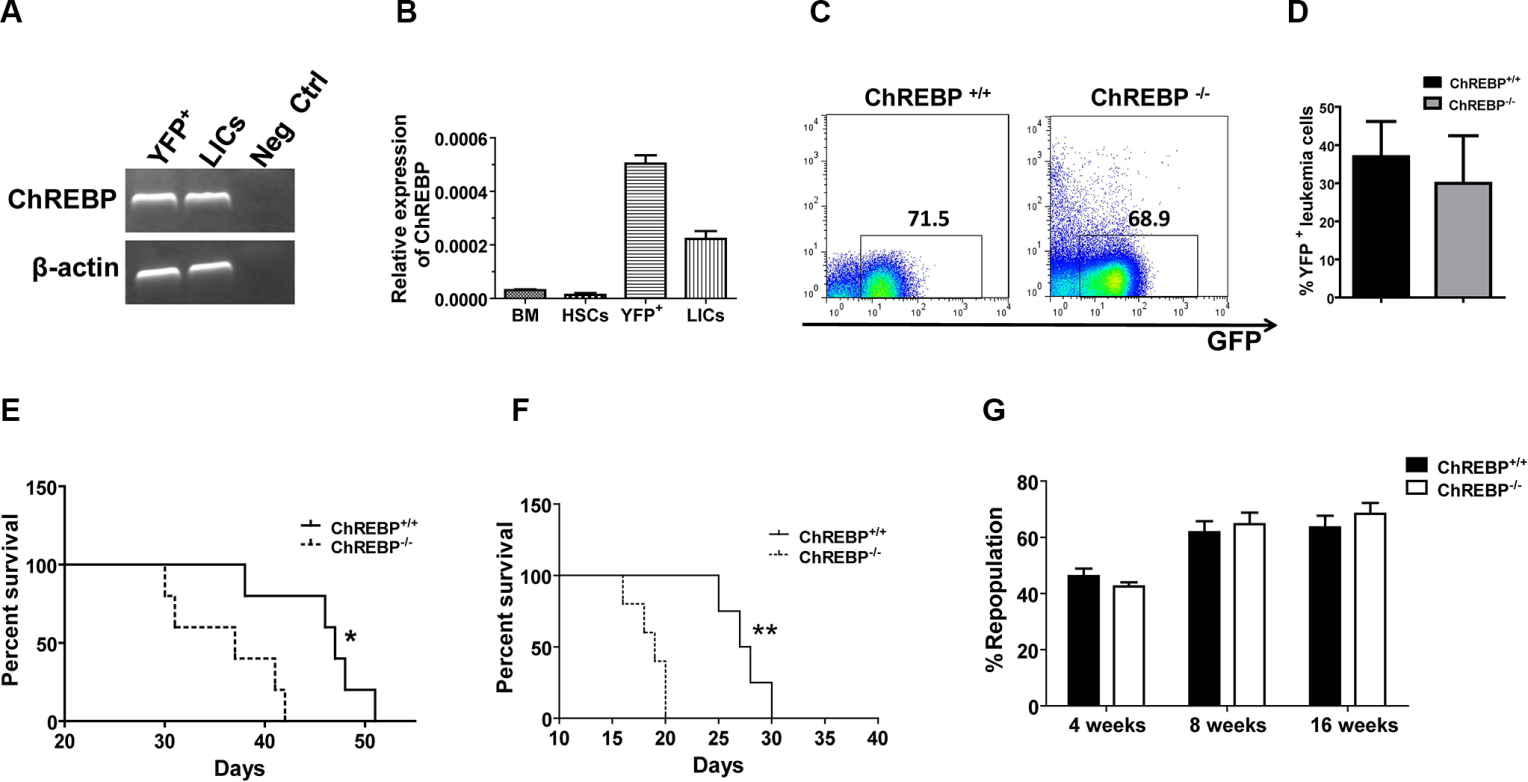
ChREBP serves as a tumor suppressor to inhibit leukemogenesis in a murine AML model (**A**) ChREBP levels in total YFP^+^ leukemia cells and YFP^+^Mac-1^+^c-Kit^+^ LICs, as determined by RT-PCR. (**B**) Quantitative RT-PCR analysis of the ChREBP levels in normal mouse bone marrow (BM) cells, Lin^−^Sca-1^+^c-Kit^+^Flk-2^−^ CD34^−^ LT-HSCs, total YFP^+^ leukemia cells and YFP^+^Mac-1^+^c-Kit^+^ LICs. (**C**) Representative flow cytometric analysis of the detection of YFP^+^ leukemia cells in the peripheral blood of the recipient mice transplanted with WT or ChREBP-null leukemia cells upon secondary transplantation. (**D**) Quantification of the frequencies of YFP^+^ cells in WT or ChREBP-null recipient mice shown in panel C (*n* = 5). (**E**) Secondary transplantation of 10,000 YFP^+^ leukemia cells resulted in the significantly reduced survival of ChREBP-null leukemia cells compared to WT cells (*n* = 5). (**F**) Comparison of the survival of recipient mice receiving WT or ChREBP-null leukemia cells upon the third transplantation (*n* = 5). (**G**) Repopulation from WT and ChREBP-null HSCs at the indicated time points. (Scale bars, 20 μm; *p < 0.05; **p < 0.01).

ChREBP has been found to be a key regulator of glycolysis and lipogenesis in hepatocytes. Interestingly, we demonstrated that ChREBP was also highly expressed in mouse YFP^+^ leukemia cells or YFP^+^Mac-1^+^c-Kit^+^ LICs from a MLL-AF9-induced AML model, as determined by RT-PCR (Figure 1A). The LICs expressed much higher levels of ChREBP compared to normal HSCs, as evaluated by quantitative RT-PCR, although their ChREBP expression level was lower than that in the total bone marrow (BM) leukemia cells (YFP^+^ cells, Figure 1B). Next, we measured the frequencies of YFP^+^ leukemia cells in the peripheral blood of WT and ChREBP-null (ChREBP^+/+^ and ChREBP^−/−^ hereafter) recipient mice (Supplementary Figure 1A–1B), which only expressed myeloid cell markers (Mac-1 and Gr-1, Supplementary Figure 1C–1D), but not lymphoid cell markers (CD3 and B220, Supplementary Figure 1E–1F). However, there were no significant differences in the percentages of YFP^+^ leukemia cells in the peripheral blood, the sizes of the leukemic spleens and livers, the YFP^+^Mac-1^+^c-Kit^+^ LIC frequencies and the survival between the recipients receiving WT and ChREBP-null cells (Supplementary Figure 1A–1B and 1G–1K), We noticed that there was a slightly increased frequency of ChREBP-null YFP^+^Mac-1^+^Gr-1^−^ leukemia cells in the peripheral blood, although the increase was not significant (Supplementary Figure 1C–1D).

To evaluate the roles of ChREBP in leukemogenesis, we conducted a secondary transplantation with WT and ChREBP-null primary leukemia cells. Although we did not observe significant changes in the frequencies of YFP^+^ leukemia cells in the peripheral blood at 5 weeks post-transplantation (Figure 1C–1D), the recipients of MLL-AF9-transduced ChREBP-null cells had a significantly reduced survival upon secondary transplantation (Figure 1E). Consistently, a subsequent third transplantation experiment also exhibited that ChREBP-null leukemic mice died much faster compared to WT controls (Figure 1F). In contrast, we revealed that ChREBP was not required for normal hematopoiesis, as determined by a competitive reconstitution analysis (Figure 1G), which indicates that ChREBP may be an ideal target for LICs. Due to the slight phenotypic changes in the primary recipient mice, we decided to focus on the phenotypes in the secondary recipient mice hereafter.

### ChREBP promotes the differentiation of LICs

**Figure 2:**
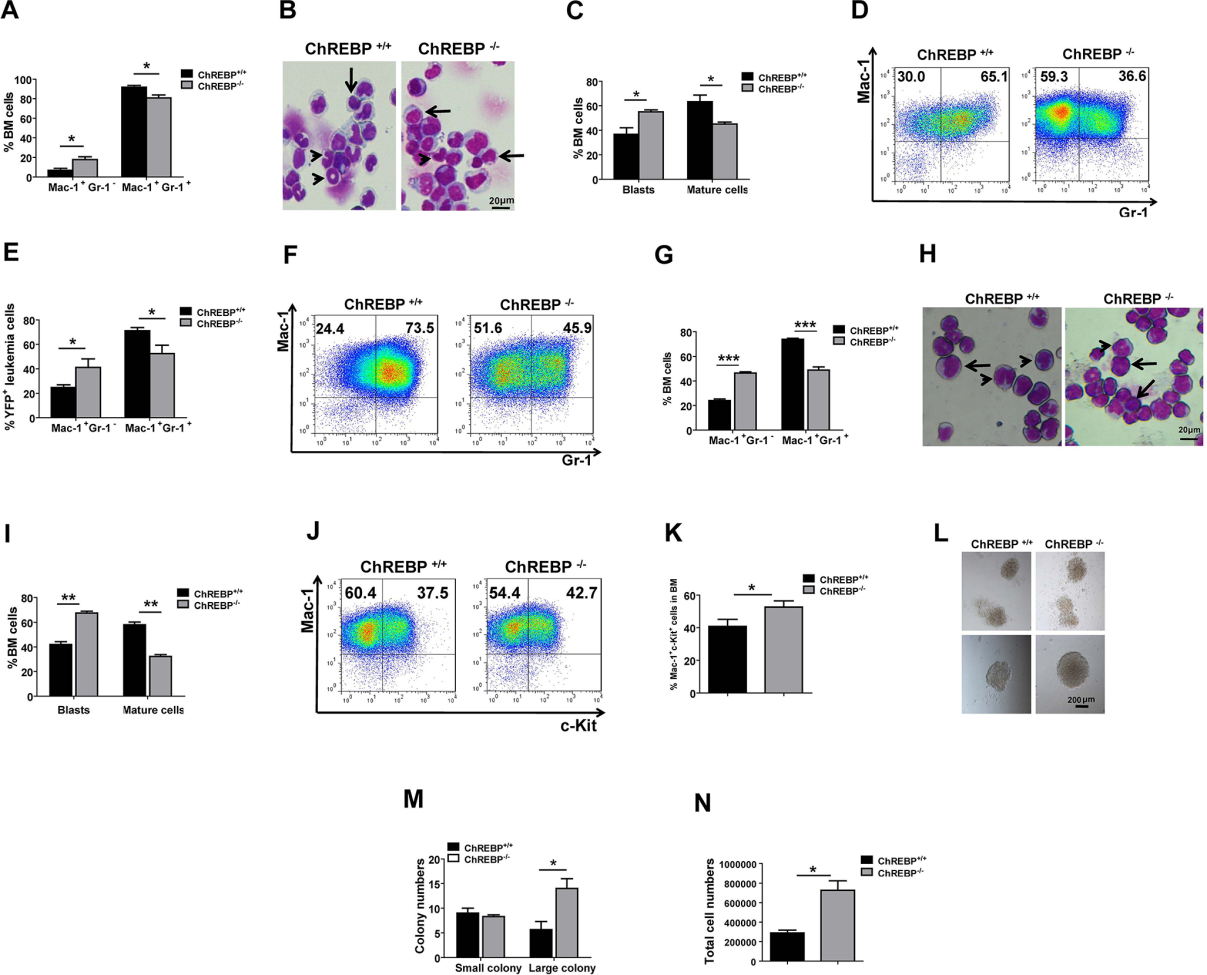
ChREBP promotes the differentiation of LICs (**A**) Quantification of the data of the YFP^+^Mac1^+^Gr1^+^ and YFP^+^Mac1^+^Gr1^−^ leukemia cells in the BM of recipients transplanted with MLL-AF9-induced WT or ChREBP-null Lin^−^ cells upon primary transplantation (*n* = 4). (**B**) Representative images of Wright-Giemsa staining of WT or ChREBP-null bone marrow leukemia cells upon primary transplantation. (**C**) Quantification of the blast cells (arrows) and differentiated cells (mature cells, arrowheads) shown in panel B. A total of 20–30 cells were counted for each section and 8–10 sections were evaluated overall (*n* = 3). (**D**) Representative flow cytometric analysis of the percentages of YFP^+^Mac1^+^Gr1^+^ and YFP^+^Mac1^+^Gr1^−^ leukemia cells in the peripheral blood of recipients transplanted with WT or ChREBP-null leukemia cells upon secondary transplantation. (**E**) Quantification of the data shown in panel D (*n* = 5). (**F**) Representative flow cytometric analysis of the percentages of YFP^+^Mac1^+^Gr1^+^ and YFP^+^Mac1^+^Gr1^−^ leukemia cells in the BM of recipients transplanted with WT or ChREBP-null leukemia cells upon secondary transplantation. (**G**) Quantification of the data shown in panel F (*n* = 5). (**H**) Representative images of Wright-Giemsa staining of WT or ChREBP-null BM leukemia cells upon secondary transplantation. (**I**) Quantification of the blast cells (arrows) and mature cells (arrowheads) shown in panel H. A total of 15–40 cells were counted for each section and 8-10 sections were evaluated overall (*n* = 3). (**J**) Representative flow cytometric analysis of the frequencies of YFP^+^Mac1^+^c-Kit^+^ LICs in the BM of recipients transplanted with WT or ChREBP-null leukemia cells upon secondary transplantation. (**K**) Quantification of the data shown in panel J (*n* = 3). (**L**) Representative images of colony-forming analysis with 1,000 WT and ChREBP-null AML cells. (**M–N**) Quantification of the results of the colony numbers and total cell numbers shown in panel L (*n* = 3). (Scale bars, 20 μm; *p < 0.05; **p < 0.01; ***p < 0.001).

To further confirm the changes in the differentiation of ChREBP-null AML cells, we first examined the frequencies of YFP^+^Mac-1^+^Gr-1^−^ leukemia cells in the BM of the mice upon primary transplantation, which was significantly increased compared to the controls (17.75 ± 2.54% vs 6.85 ± 1.72%, Figure 2A). This change in the Gr-1 expression levels, which represent the extent of myeloid differentiation, indicated that differentiation was blocked in ChREBP-null leukemia cells. Wright-Giemsa staining further revealed that many more immature blast cells appeared in ChREBP-null recipients than in WT counterparts (Figure 2B–2C). Moreover, there was an approximately 2-fold higher frequency of YFP^+^Mac-1^+^Gr-1^−^ leukemic cells in both the peripheral blood (Figure 2D–2E) and the BM (Figure 2F–2G) of ChREBP-null recipients upon secondary transplantation. This was consistent with the more immature blast cells found in the recipients of ChREBP-null leukemia cells (Figure 2H–2I).

Meanwhile, the phenotypic LIC frequencies in the recipients of ChREBP-null leukemia cells were markedly increased (Figure 2J–2K). An *in vitro* functional assay using colony-forming units further demonstrated that there were more large colonies (diameter > 500 μm) and a notably increased cell number in colonies derived from ChREBP-null leukemia cells isolated from secondary recipients, indicating their enhanced clonogenic potential (Figure 2L–2N). The apoptotic status of LICs, as analyzed by Annexin V/7-AAD staining, exhibited no significant differences (Supplementary Figure 2A–2B). Finally, no detectable changes were found in the cell cycle, as determined by staining with either Ki-67/Hoechst 33342 (Supplementary Figure 2C–2D) or an *in vivo* BrdU incorporation assay (Supplementary Figure 2E–2F). These results suggest that ChREBP may contribute to enhanced LIC differentiation, a decreased LIC pool and delayed leukemogenesis.

### ChREBP controls the differentiation of LICs through TXNIP

**Figure 3:**
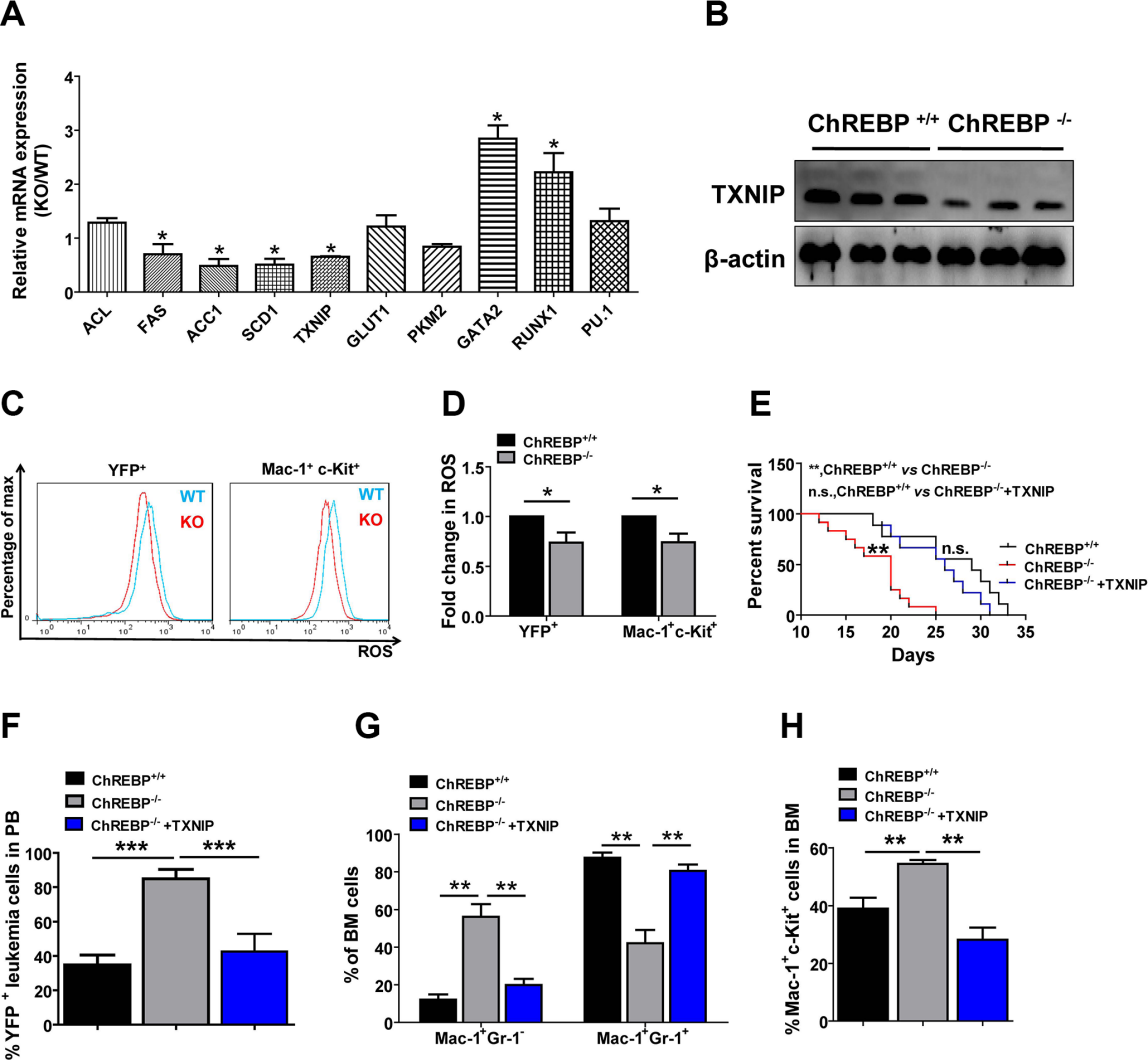
ChREBP controls the differentiation of LICs through TXNIP (**A**) Quantitative RT-PCR analysis of the potential candidate genes related to lipogenesis, glycolysis and myeloid differentiation in FACS-purified YFP^+^Mac-1^+^c-Kit^+^ WT or ChREBP-null LICs (*n* = 3). (**B**) The expression levels of TXNIP were measured by immunoblotting in WT and ChREBP-null LICs (*n* = 3). (**C**) Representative flow cytometric analysis of the ROS levels in WT and ChREBP-null YFP^+^ leukemia cells or YFP^+^Mac-1^+^c-Kit^+^ LICs using 5-(and-6)-carboxy-2′,7′-dichlorofluorescein diacetate. (**D**) Quantification of the results described in panel C (*n* = 3). (**E**) TXNIP was overexpressed in ChREBP-null leukemia cells and transplanted into the recipient mice. Survival was analyzed among the mice receiving WT leukemia cells, ChREBP-null leukemia cells and TXNIP-overexpressing ChREBP-null leukemia cells, respectively (*n* = 9–11, log-rank test). (**F–H**) Frequencies of YFP^+^ leukemia cells in the peripheral blood, and YFP^+^Mac1^+^Gr1^+^/Gr1^−^ leukemia cells and YFP^+^Mac-1^+^c-Kit^+^ LICs in the BM were measured in the recipient mice shown in panel E. (*p < 0.05; **p < 0.01; ***p < 0.001).

Because ChREBP has been well known to be involved in glycolysis and lipogenesis in hepatocytes and may be involved in the regulation of differentiation in LICs, as reported here, we next tried to identify the potential targets related to the cells' phenotypes. Surprisingly, we did not find notable changes in several glycolysis-related genes (GLUT1, PKM2), as measured by quantitative RT-PCR in ChREBP-null LICs (Figure 3A). Consistently, the ATP level and lactate production, which are indicative of glycolysis (extracellular acidification rate, ECAR), remained unchanged, as determined with the Seahorse XF96 extracellular flux analyzer (Supplementary Figure 3A–3B). However, RUNX1 and GATA2 (but not PU.1), which are two critical transcription factors for the inhibition of differentiation, were dramatically increased upon ChREBP deletion (Figure 3A).

Moreover, several known targets important for lipogenesis, including FAS, ACC1, SCD1 and TXNIP (but not ACL), were markedly downregulated in ChREBP-null LICs. Many studies indicate that lipogenesis is required for the growth of cancer cells, which contradicts the marked decrease of FAS, ACC1 and SCD1 (genes that enhance lipogenesis) and accelerated leukemia development upon ChREBP deletion reported here. Interestingly, we found that TXNIP (a critical gene that inhibits lipogenesis [5]) was downregulated in ChREBP-null LICs. TXNIP has been reported to be involved in many cellular and physiological processes in addition to its function in the negative regulation of lipogenesis [22, 23]. For example, TXNIP can serve as an inhibitor for the activity of thioredoxin [4, 23], a mediator of glucose metabolism [5, 25], a tumor suppressor in T-cell leukemia or other cancers [26–28] or a critical regulator of the differentiation of natural killer cells [29]. Taken together, all these clues led us to speculate that TXNIP may be a potential target of ChREBP to suppress leukemia development.

To ask whether TXNIP regulates the differentiation of LICs, we further evaluated TXNIP expression levels by western blotting and demonstrated that the TXNIP levels were strikingly reduced in ChREBP-null LICs (Figure 3B). Because the increased expression of TXNIP may lead to enhanced ROS levels, which is a potent driver of differentiation [30], we measured the ROS levels in leukemia cells by staining with DCFDA. Consistently, both ChREBP-null YFP^+^ BM leukemia cells and LICs had relatively lower ROS levels compared to the WT controls (Figure 3C–3D). To confirm whether TXNIP is a direct downstream target for ChREBP, we overexpressed TXNIP in ChREBP-null leukemia cells and transplanted them into recipient mice. Our results clearly displayed that the mice transplanted with the TXNIP-overexpressing, ChREBP-null AML cells developed leukemia much more slowly than those transplanted with the ChREBP-null control cells, which were comparable to their WT counterparts (Figure 3E). Meanwhile, the overexpression of TXNIP efficiently rescued the phenotypes in ChREBP-null leukemic mice, as shown by the decreased frequencies of YFP^+^ peripheral blood leukemia cells, YFP^+^Mac-1^+^GR-1^−^undifferentiated leukemia cells, and YFP^+^Mac-1^+^c-Kit^+^ LICs (Figure 3F–3H) as well as the increased percentages of mature leukemia cells in the BM (Supplementary Figure 4A–4B). Consistently, RUNX1, but not GATA2, was remarkably upregulated in ChREBP-null leukemia cells, which could be partially suppressed by the ectopic expression of TXNIP (Supplementary Figure 4C). Collectively, these results indicate that ChREBP suppresses the leukemogenic potential of AML cells through TXNIP, which may contribute to the enhanced differentiation of LICs through RUNX1.

### ChREBP transactivates TXNIP to enhance differentiation by inhibiting RUNX1

**Figure 4:**
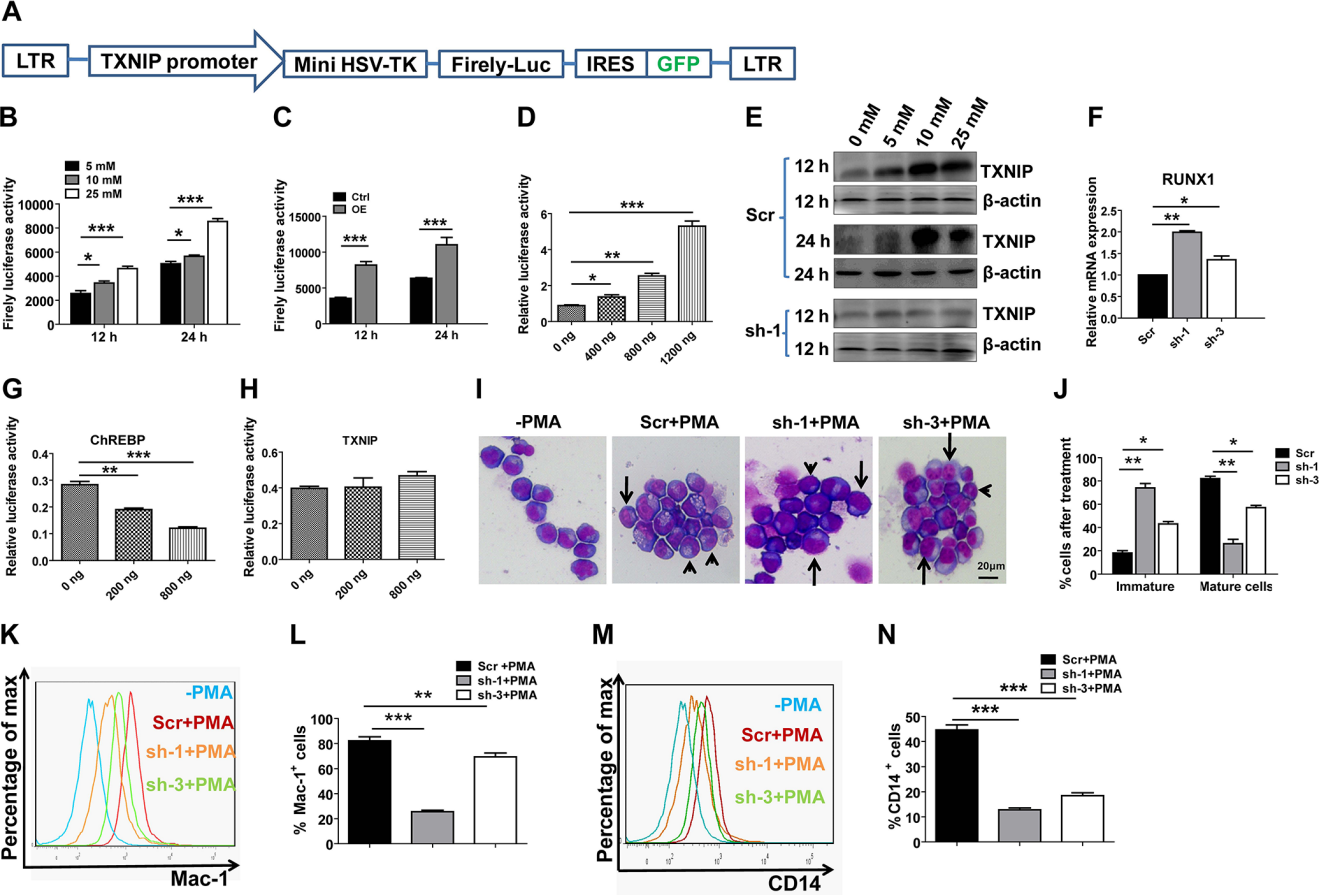
ChREBP transactivates TXNIP to regulate the differentiation of LICs through the downregulation of RUNX1 (**A**) Schematic diagram of the TXNIP lentiviral reporter vector (plenti-TXNIP-GFP). (**B**) Luciferase reporter assays demonstrated a dose-dependent transcriptional activation of TXNIP by different concentrations of glucose (*n* = 3). (**C**) Luciferase activity was measured in THP1 cells containing a TXNIP lentiviral reporter at the indicated time points following ChREBP overexpression (OE). (**D**) Luciferase activity was measured in 293T cells cotransfected with a TXNIP lentiviral reporter and different amounts of ChREBP. (**E**) TXNIP expression levels were determined in THP1 cells subjected to knockdown with scrambled shRNA or ChREBP-targeting shRNA (sh-1) by western blotting at the indicated time points during glucose treatment. (**F**) Relative mRNA levels of RUNX1 in THP1 cells following ChREBP knockdown by shRNAs (sh-1 or sh-3) (*n* = 3). (**G–H**) Luciferase activity was measured in 293T cells cotransfected with a RUNX1 reporter and different amounts of ChREBP or TXNIP. (**I**) Representative images of Wright-Giemsa staining of THP1 cells treated with the scrambled or ChREBP-targeting shRNA (sh-1 and sh-3) following PMA treatment for 48 h. Undifferentiated cells (arrows) and differentiated cells (arrowheads) are indicated. (**J**) Quantification of the results described in panel I.A total of 10–30 cells were counted for each section and 8–10 sections were evaluated overall. (*n* = 3). (**K–M**) Representative flow cytometric analysis of the levels of Mac-1 (K–L) or CD14 (**M–N**) on THP1 cells treated with the scrambled or ChREBP-targeting shRNA (sh-1 and sh-3) following PMA treatment for 48 h. The quantification of the data is also shown (*n* = 3). (Scale bars, 20 μm; *p < 0.05; **p < 0.01; ***p < 0.001).

To figure out how TXNIP is regulated by ChREBP, we first constructed a lentiviral luciferase reporter (plenti-TXNIP-GFP) containing a conserved ChREBP-binding site (Figure 4A) and demonstrated a dose-dependent activation of TXNIP in THP1 cells with exogenous supplementation of glucose (glucose can effectively induce ChREBP activation, as reported previously) (Figure 4B). ChREBP also directly bound to the promoter of TXNIP, as demonstrated by chromatin Immunoprecipitation (ChIP) assay in THP1 cells (Supplementary Figure 5A), or as determined using a luciferase reporter in THP1 or 293T cells (Figure 4C–4D). We further confirmed the activation of TXNIP by detecting its protein levels by western blotting at 12 h and 24 h post-treatment (Figure 4E). In contrast with the above data, the ChREBP-mediated transactivation of TXNIP was totally abrogated when ChREBP was knocked down in THP1 cells (Supplementary Figure 5B and Figure 4E). Similar to our results in ChREBP-null mice, knockdown of ChREBP with specific shRNAs in THP1 cells led to a significant upregulation of RUNX1 (Figure 4F). Interestingly, we also found that ChREBP, but not TXNIP, directly bound to the RUNX1 promoter, as determined using a luciferase assay (Figure 4G–4H). However, neither ChREBP nor TXNIP bound to the GATA2 promoter (Supplementary Figure 5C–5D). These results suggest that ChREBP mainly collaborates with TXNIP and RUNX1 to mediate their function in LIC differentiation.

To further evaluate whether ChREBP has a similar effect on the differentiation of THP1 cells as that in mouse LICs, we knocked down ChREBP in THP1 cells and examined the morphologic changes after treatment with PMA, which can potently induce the differentiation of THP1 cells. As shown in Figure 4I–4J, much lower frequencies of differentiated THP1 cells were observed in ChREBP-knockdown cells, indicating that ChREBP promotes differentiation in myeloid leukemia cells. Consistently, levels of Mac-1 and CD14, two markers for myeloid differentiation, were remarkably decreased in ChREBP-knockdown THP1 cells upon PMA treatment (Figure 4K–4N). We also noticed that the downregulation of ChREBP in THP1 cells resulted in a notable expansion *in vitro*, even two days after knockdown (Supplementary Figure 5E). Similar effects were observed in other ChREBP-knockdown leukemia cell lines, including U937 and HL60 (Supplementary Figure 5F–5G). In contrast, the overexpression of ChREBP caused a marked delay in the growth of THP1 cells (Supplementary Figure 5H–5I).

### TXNIP suppresses the proliferation and promotes the differentiation of a mouse leukemia cell line

**Figure 5:**
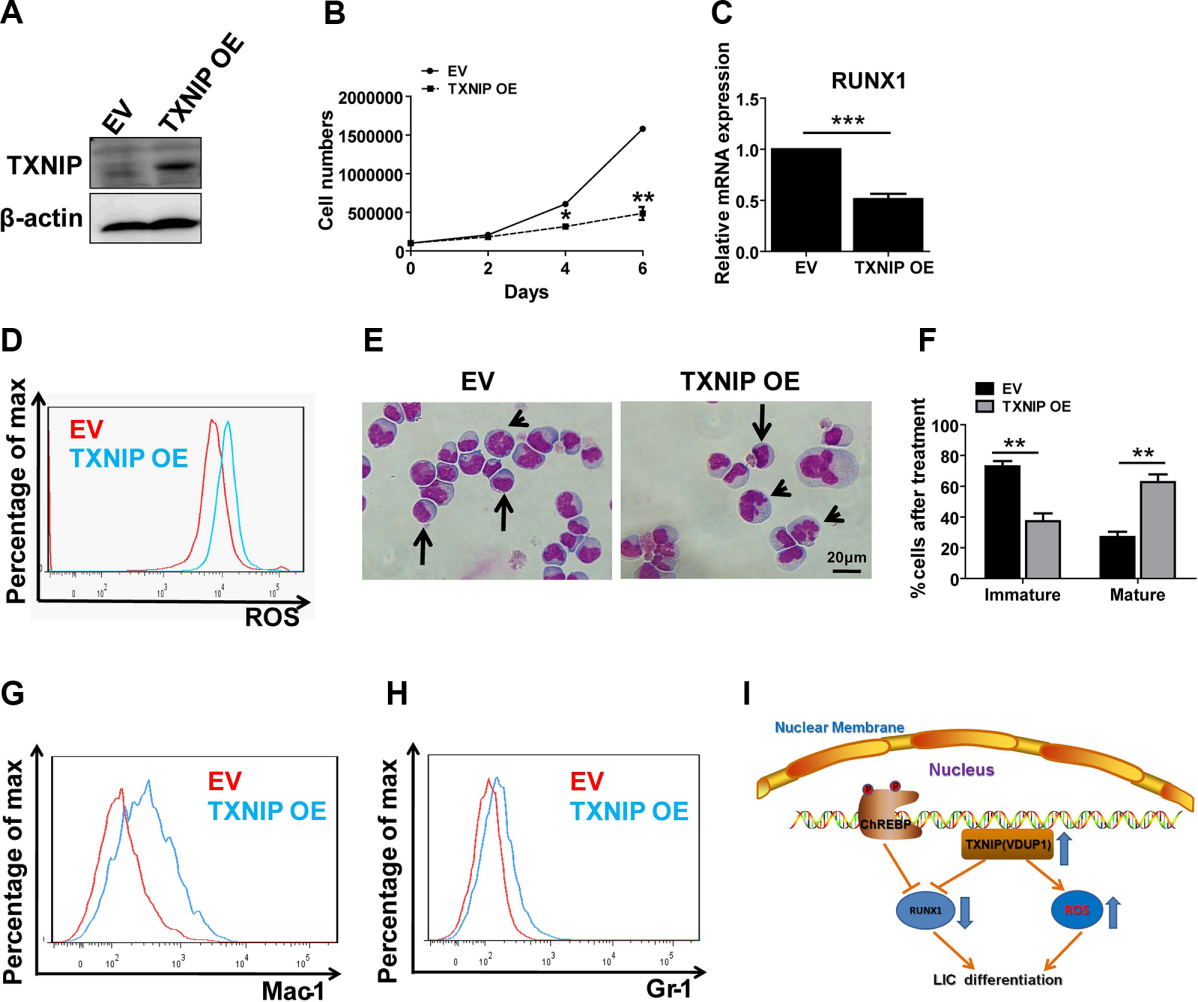
TXNIP promotes differentiation in a mouse leukemia cell line and suppresses its proliferation (**A–B**) Overexpression (OE) of TXNIP in C1489 cells was measured by immunoblotting analysis (A) and the cell numbers were calculated at indicated time points (B). (**C**) Relative mRNA expression levels of RUNX1 in control or TXNIP-overexpressing C1498 cells. (**D**) Representative flow cytometric analysis of ROS levels in control and TXNIP-overexpressing C1498 cells measured by staining with 5-(and-6)-carboxy-2′,7′-dichlorofluorescein diacetate. (**E**) Representative images of Wright-Giemsa staining of control or TXNIP-overexpressing C1498 cells following PMA treatment for 48 h. Immature cells (arrows) and mature cells (arrowheads) are indicated. (**F**) Quantification of the results described in panel E. A total of 15–20 cells were counted for each section and 8–10 sections were evaluated overall (*n* = 3). (**G–H**) Representative flow cytometric analysis of the levels of Mac-1 or Gr-1 in control or TXNIP-overexpressing C1498 cells. (**I**) Schematic diagram for the functions of ChREBP in leukemogenesis. ChREBP inhibits leukemia development by transactivating TXNIP, which downregulates the expression levels of RUNX1, or directly suppresses the RUNX1 expression to promote myeloid differentiation. (Scale bars, 20 μm; *p < 0.05; **p < 0.01; ***p < 0.001).

To ask whether TXNIP has a direct impact on the proliferation of leukemia cells, we overexpressed TXNIP in C1498 cells, which is a myeloid leukemia cell line, and demonstrated that the overexpression of TXNIP dramatically inhibited the expansion of C1498 cells *in vitro* (Figure 5A–5B). As expected, RUNX1 was significantly downregulated and the ROS level was slightly increased upon TXNIP overexpression (Figure 5C–5D). TXNIP-overexpressing C1498 cells were prone to differentiation, as shown by Wright-Giemsa staining (Figure 5E–5F). Flow cytometric analysis also showed a notably increased percentage of Mac-1^+^ or Gr-1^+^ differentiated cells (Figure 5G–5H). These data suggest that TXNIP enhances the differentiation of leukemia cells. In summary, as shown in the working model (Figure 5I), ChREBP directly suppresses RUNX1 expression or transactivates TXNIP to further downregulate the expression of RUNX1 and enhance the ROS levels to promote the differentiation of LICs, which remarkably delays leukemogenesis.

## DISCUSSION

In this study, we provide intriguing evidence that ChREBP serves as a tumor suppressor, rather than as an oncogene, in leukemia development. This is similar to some other transcription factors, such as EZH2, which can function as both a tumor suppressor and an oncogene in different types of cancers [31–33]. A genome-wide analysis of ChREBP targets also reveals that ChREBP may act as either a transcriptional repressor or an activator [34].

In this study, we showed that ChREBP was highly upregulated in YFP^+^ leukemia cells and LICs compared to normal BM cells and HSCs, indicating that ChREBP served as an oncogene. However, we also noticed that the ChREBP expression level was lower in LICs compared to the total YFP^+^ leukemia cells, which suggests that ChREBP may be a tumor suppressor. Thus far, most evidence indicates that ChREBP acts as an oncogene in many solid tumors, such as liver and colon cancers [19]. However, very few reports also indicate that a high expression level of ChREBP may be related with the increased survival of patients with certain types of breast cancers, indicating its inhibitory role in some tumors [35], which is similar to what we reported here. Currently, we do not find any reports related to the relationship between ChREBP mutations and human cancers. An in-depth analysis and more evidence are required to confirm the differential roles of ChREBP in different types of cancers.

ChREBP has been reported to be involved in many physiological or pathological activities, including glycolysis, lipogenesis, amino acid metabolism and cell motility [36–40]. TXNIP, a critical downstream target of ChREBP and a negative regulator of lipogenesis, has numerous functions in glucose and lipid metabolism, redox status and NK cell differentiation. Although we currently do not have much evidence to show that lipogenesis may play a critical role in leukemogenesis through TXNIP, we demonstrated that TXNIP could enhance ROS generation and downregulate the expression levels of RUNX1 to promote the differentiation of LICs. It has been suggested that ROS levels fine-tune the cell fates of many tumors. Low levels of ROS may be required for cell proliferation, differentiation and survival, whereas excessively high levels of ROS may lead to enhanced oxidative stress and cell death. Our study suggests that ChREBP transactivates TXNIP and enhances the production of ROS to promote leukemia differentiation and delay leukemogenesis, although the detailed mechanisms await further investigation. To our knowledge, this is the first body of evidence showing that ChREBP/TXNIP/RUNX1 signaling plays an essential role in the suppression of leukemogenesis. We also speculate that some other metabolic genes, similar to ChREBP, may have dual functions in the initiation, development or relapse of different types of tumors.

RUNX1 and GATA2 have been considered two critical transcription factors for stemness of HSCs [41–44]. The levels of RUNX1 and GATA2 are fine-tuned during hematopoiesis as well as leukemogenesis. Interestingly, there exist dose effects of both RUNX1 and GATA2 in leukemia development because either too low or too high levels of these proteins significantly influence the initiation, maintenance and relapse of leukemia [45]. Notably, our data indicate that ChREBP directly binds to the RUNX1 promoter, but not to the GATA2 promoter, to promote the differentiation of LICs. TXNIP can further suppress RUNX1, but not GATA2, expression. Meanwhile, both ChREBP and TXNIP effectively inhibit the mRNA levels of RUNX1 and GATA2, although the underlying mechanism remains largely unknown. In summary, we provide interesting and important clues showing that ChREBP may play differential roles in different types of cancers. ChREBP acts as a tumor suppressor to promote the differentiation of LICs but does not promote the differentiation of normal HSCs. ChREBP and its downstream molecules, including TXNIP, RUNX1 and GATA2, may be ideal therapeutic targets for certain types of leukemia.

## MATERIALS AND METHODS

### Mice

The ChREBP-null mice were kindly provided by Dr. Xuemei Tong at Shanghai Jiao Tong University School of Medicine. C57BL/6 CD45.2 mice were ordered from the Shanghai SLAC Laboratory Animal Co. Ltd. Animal experiments were approved and conducted according to the Guidelines for Animal Care at Shanghai Jiao Tong University School of Medicine.

### Leukemia cell lines

Several mouse or human AML cell lines, including C1498 (mouse), THP1 (human, M5), HL60 (human, M3) and U937 (human, M5) were cultured in RPMI 1640 medium (Hyclone) supplemented with 10% FBS (Hyclone). All the cell lines were from ATCC.

### Retroviral infection and transplantation

The MSCV-MLL-AF9-IRES-YFP-encoding plasmid [46] and the packaging plasmid pCL-ECO were used to co-transfect 293T cells using the calcium phosphate transfection method. MLL-AF9-expressing retroviruses were collected 48–72 h after transfection. Lin^−^ fetal liver cells were isolated from WT and ChREBP-null mice and infected with MLL-AF9 retroviruses by spinoculation in the presence of 4 μg/mL Polybrene. The cells were cultured overnight in DMEM with 10% FBS, 20 ng/mL SCF, 20 ng/mL IL-3 and 10 ng/mL IL-6, followed by another round of spin infection. The infected cells (300,000) were transplanted into lethally irradiated (1,000 rad) C57BL/6 mice by retro-orbital injection. YFP^+^ bone marrow leukemia cells from primary transplanted mice were further isolated and injected into recipient mice for serial transplantation. For the rescue experiment, the retroviral plasmid MSCV-TXNIP-IRES-mCherry was used to transfect 293T cells, and the resulting retroviral supernatant was collected for spin infection with ChREBP-null leukemia cells, followed by retro-orbital injection into recipient mice.

### Flow cytometry

Peripheral blood was collected by retro-orbital bleeding, and bone marrow cells were isolated from the femurs and tibias of leukemic mice. Flow cytometry and cell cycle analyses were performed as we described previously [47]. Briefly, leukemia cells were stained with anti-mouse Mac-1-APC, anti-mouse Gr-1-PE, anti-mouse CD3-APC, anti-mouse B220-PE or anti-mouse c-Kit-PE monoclonal antibodies (eBioscience). The cell cycle stages were evaluated with either Ki-67/7-AAD staining (BD Pharmingen) or a 5-bromo-2′-deoxyuridine (BrdU) incorporation assay. For the analysis of apoptosis, leukemia cells were stained with PE-conjugated anti-Annexin V and 7-AAD (BD Pharmingen) according to the manufacturer's protocol. For the measurement of ROS, the cells were incubated with 1 μM 5-(and-6)-carboxy-2′,7′-dichlorofluorescein diacetate (carboxy-DCFDA, Invitrogen) for 30 minutes at 37°C, followed by flow cytometric analysis. For the examination of the BrdU incorporation assay, leukemic mice were subjected to three intraperitoneal injections of BrdU (Sigma; 3 mg/24 hours) in PBS. The BM cells were fixed, permeabilized and denatured, followed by antibody staining with anti–BrdU-APC according to the manufacturer's instructions (BD Pharmingen).

### Western blotting

Equal numbers of bone marrow leukemia cells or leukemia cell lines were collected for further immunoblotting. Whole cell lysates were electrophoresed on 8–10% sodium dodecyl sulfate polyacrylamide gels and transferred onto polyvinylidene difluoride membranes (Millipore). The membranes were blocked with 5% non-fat milk/TBS and incubated with primary antibodies at 4°C overnight. The following antibodies were used for blotting: anti-TXNIP (Proteintech), anti-ChREBP (Nova Biologicals), anti-β-actin (Sigma), anti-HA (Sigma) and anti-StrepII (Genescript).

### Differentiation analysis of THP1 or C1498 cells

THP1 cells infected with shRNA targeting ChREBP were treated with 100 ng/mL of phorbol 12-myristate 13-acetate (PMA) for 48 h as previously described [48]. Myeloid differentiation was monitored by flow cytometric analysis with antibodies against human Mac-1 and CD14 (eBiosciences) or Wright-Giemsa staining. In the other experiment, a mouse myeloid leukemia cell line, C1498 (kindly provided by Fubin Li at Shanghai Jiao Tong University School of Medicine), was induced with PMA for differentiation, followed by examination with Wright-Giemsa staining and flow cytometric analysis.

### Lentivirus construction, infection and cell proliferation assays

The lentiviral vector GIPZ was used to express shRNAs designed to target ChREBP (sequences listed in Supplementary Table 1). Using the calcium phosphate transfection method, lentivirus constructs together with the packaging plasmids pSPAX2 and pMD2G (4:3:1) were mixed and transfected into 293T cells. The supernatant, which contained lentiviruses, was harvested 48 h and 72 h later. Lentiviruses were used for subsequent infections of leukemia cell lines, including THP1, U937 and HL60 cells. Cells (200,000) treated with shRNAs targeting ChREBP or a scrambled control were cultured in 12-well plates and counted at the indicated time points.

### Quantitative RT-PCR and colony-forming unit assays

Total bone marrow cells, HSCs, YFP^+^ leukemia cells or YFP^+^Mac-1^+^c-Kit^+^ LICs were sorted by flow cytometry for the isolation of total RNA. First-strand cDNA was reverse transcribed using M-MLV reverse transcriptase (Promega Inc.). The PCR reactions were performed according to the manufacturer's protocol. The mRNA level was normalized to the level of the β-actin RNA transcripts. The primer sequences used are shown in Supplementary Table 1. For the colony-forming unit assays, the indicated numbers of cells from AML mice were plated in methylcellulose (M3534, Stem Cell Technologies) according to the manufacturer's instructions. The numbers of colonies were calculated 8–10 days after culture.

### Glycolysis assays

The ATP content was determined using an ATP Bioluminescence Assay Kit HS II (Roche) according to the manufacturer's protocols. Lactate generation was measured using the Seahorse XF96 extracellular flux analyzer as previously described, with minor modifications [49]. Briefly, three replicate wells of 3 × 10^5^ WT or ChREBP-null AML cells per well were seeded in 96-well XF96 plates coated with BD Cell-Tak (BD Biosciences) in unbuffered DMEM and incubated at 37°C for pH stabilization. Analyses were performed both under basal conditions and after the injection of oligomycin (2 μM), glucose (10 mM) and 2-DG (100 mM).

### Luciferase reporter assays

The transcriptional activation of TXNIP by ChREBP was evaluated using a constructed lentiviral luciferase reporter vector (kindly provided by Guoqiang Chen at Shanghai Jiao Tong University School of Medicine), plenti-TXNIP-GFP, which contains the conserved ChREBP-binding site, as previously described [50]. THP1 cells were infected with plenti-TXNIP-GFP lentiviruses, FACS-purified, and treated with 5, 10 and 25 mM glucose for 12 h or 24 h. Following the incubation, the luciferase activity was measured using a luciferase reporter system (GloMax^®^ Multi Instrument). Alternatively, the luciferase activity of THP1 cells or 293T cells overexpressing ChREBP and plenti-TXNIP-GFP was measured. To evaluate the transcriptional activation of RUNX1 and GATA2 by ChREBP or TXNIP, the promoter regions of RUNX1 and GATA2 were incorporated into a pGL4 vector and analyzed with a luciferase reporter system as described above.

### Chromatin immunoprecipitation assays

Chromatin immunoprecipitation (ChIP) assays were performed as described previously [51]. A total of 5 × 10^6^ ChREBP-overexpressing (with a StrepII tag) THP1 cells were collected and cross-linked by adding formaldehyde at a final concentration of 1%. The samples were sonicated with a Q700 sonicator six times (5 sec on and 10 sec off for each round of sonication). ChREBP was immunoprecipitated with Strep-Tactin beads (IBA). DNA fragments were purified using a Qiagen PCR purification kit and quantified by semi-quantitative PCR with primers for the TXNIP promoter, listed in Supplementary Table 1.

### Statistics

The data are expressed as the mean ± SEM. The data were analyzed with Student's *t* test, and the statistical significance was set at *p* < 0.05. The survival rates of the two groups were analyzed using a log-rank test.

## SUPPLEMENTARY MATERIALS FIGURES AND TABLES


