# Supplementary material for: ChREBP promotes the differentiation of leukemia-initiating cells to inhibit leukemogenesis through the TXNIP/RUNX1 pathways
Source: Oncotarget. 2016 May 20;7(25):38347–58. doi: 10.18632/oncotarget.9520 (PMC5122394; doi:10.18632/oncotarget.9520)
Supplement: Supplementary file 1 [file oncotarget-07-38347-s001.pdf]

ChREBP promotes the differentiation of leukemia-initiating cells to inhibit leukemogenesis through the TXNIP/RUNX1 pathways

Supplementary Materials

Supplementary Table S1: Primer sequences

Gene	Forward (5′–3′)	Reverse (5′–3′)
Mouse ACL	TGTTCTTGGTCAGCTTTGTAGC	AGGCTGTGGGTCTTGTTTAGG
Mouse FAS	TTTGCTGCCGTGTCCTTCTACC	ATGTGCACAGACACCTTCCCGT
Mouse ACC1	GATGAACCATCTCCGTTGGC	GACCCAATTATGAATCGGGAGTG
Mouse SCD1	CACCTGCCTCTTCGGGATTT	ACGTCATTCTGGAACGCCAT
Mouse TXNIP	GTCAGTGTCCCTGGCTCCAAGA	AGCTCATCTCAGAGCTCGTCCG
Mouse GLUT1	TCTCGGCTTAGGGCATGGAT	TCTATGACGCCGTGATAGCAG
Mouse PKM2	GTGGCTCGGCTGAATTTCTCT	CACCGCAACAGGACGGTAG
Mouse GATA2	CGACGAGGTGGATGTCTTCT	GCTGTGCAACAAGTGTGGTC
Mouse RUNX1	GATGGCACTCTGGTCACCG	GCCGCTCGGAAAAGGACAA
Mouse PU.1	ATGTTACAGGCGTGCAAAATGG	TGATCGCTATGGCTTTCTCCA
Mouse ChREBP	ATCAGCGCTTTGACCAGATG	GGGAATTCAGGACAGTTGGC
Mouse β-actin	GGCTGTATTCCCCTCCATCG	CCAGTTGGTAACAATGCCATGT
Human RUNX1	AACCCAGCATAGTGGTCAGC	GGCATCGTGGACGTCTCTAG
Human GATA2	GCTGCACAATGTTAACAGGC	TCTCCTGCATGCACTTTGAC
Human ChREBP	AAGATCCGCCTGAACAACG	CACTTGTGGTATTCCCGCATC
Human β-actin	AGAGCTACGAGCTGCCTGAC	AGCACTGTGTTGGCGTACAG
TXNIP (for ChIP)	CTCGCGTGGCTCTTCTG	GCAGGAGGCGGAAACGT
**ShRNA for ChREBP**	**Targeted sequence(5′–3′)**	
shRNA1 (sh-1)	CGAGACATGTTTGATGACT	
shRNA2 (sh-2)	CGTCCAGACAGCAACAAGA	
shRNA3 (sh-3)	CGCAGGAGACAGTCCCTGA	

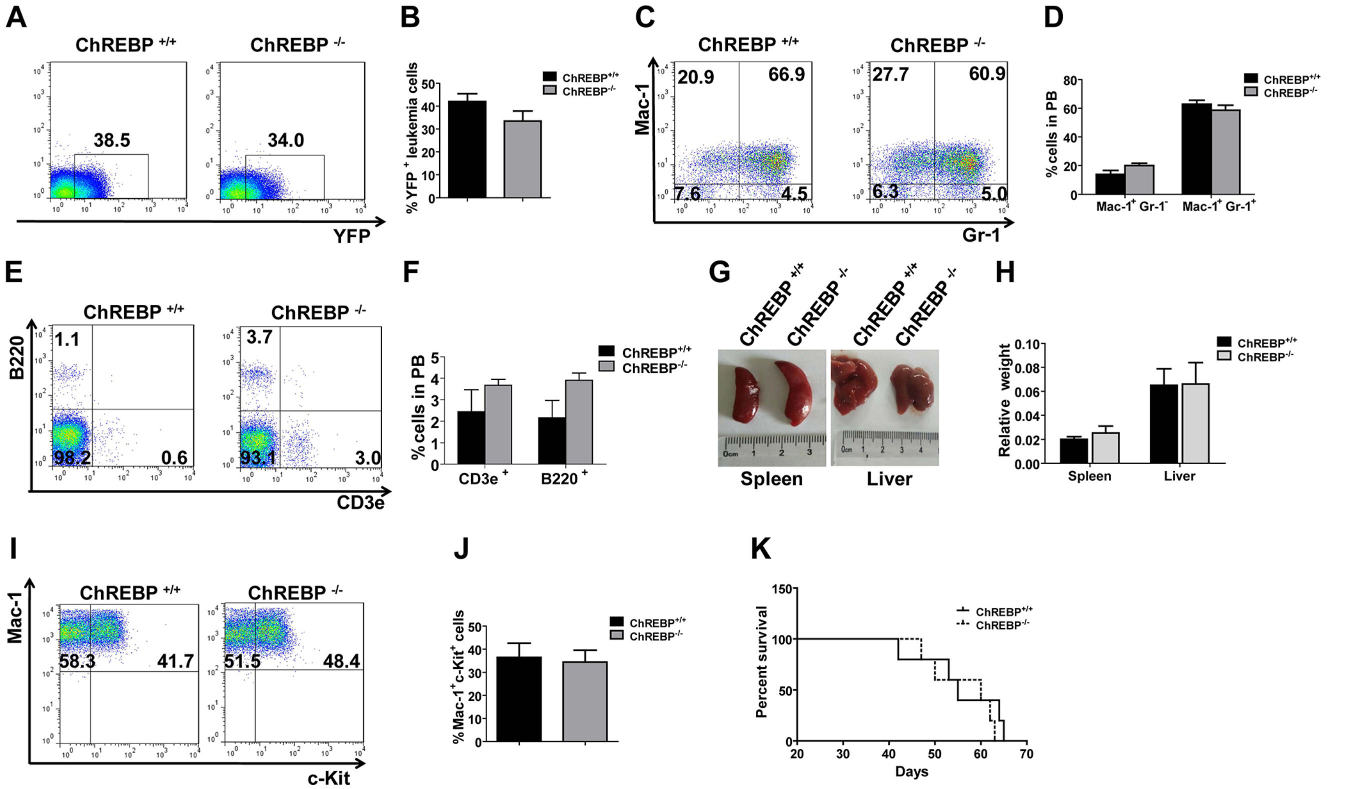

Supplementary Figure S1: Characteristics of ChREBP-null mice. (**A**) Representative flow cytometric analysis of the frequencies of YFP+ leukemia cells in the peripheral blood of recipient mice transplanted with MLL-AF9-induced WT or ChREBP-null Lin- cells upon primary transplantation. (**B**) Quantification of the results shown in panel A (n = 5). (**C**) Representative flow cytometric analysis of the percentages of $YFP^+Mac1^+Gr1^+$ and $YFP^+Mac1^+Gr1^-$ leukemia cells in the peripheral blood of recipient mice transplanted with MLL-AF9-infected WT or ChREBP-null fetal liver cells upon primary transplantation. (**D**) Quantification of the data shown in panel C (n = 5). (**E**) Representative flow cytometric analysis of the percentages of YFP^+CD3e^+ or YFP^+B220^+ cells in the peripheral blood of recipient mice transplanted with MLL-AF9-induced WT or ChREBP-null fetal liver cells upon primary transplantation. (**F**) Quantification of the percentages of leukemia cells shown in panel E (n = 4). (**G**) Comparison of the liver and spleen sizes of mice that were transplanted with WT or ChREBP-null MLL-AF9-induced fetal liver cells upon primary transplantation. (**H**) Quantification of the relative weights of the livers and spleens shown in panel G (n = 3). (**I**) Representative flow cytometric analysis of the frequencies of WT or ChREBP-null LICs upon primary transplantation. (**J**) Quantification of the data shown in panel I (n = 5). (**K**) Survival analysis of the mice transplanted with MLL-AF9 induced WT or ChREBP-null Lin- cells upon primary transplantation (n = 5, log-rank test).

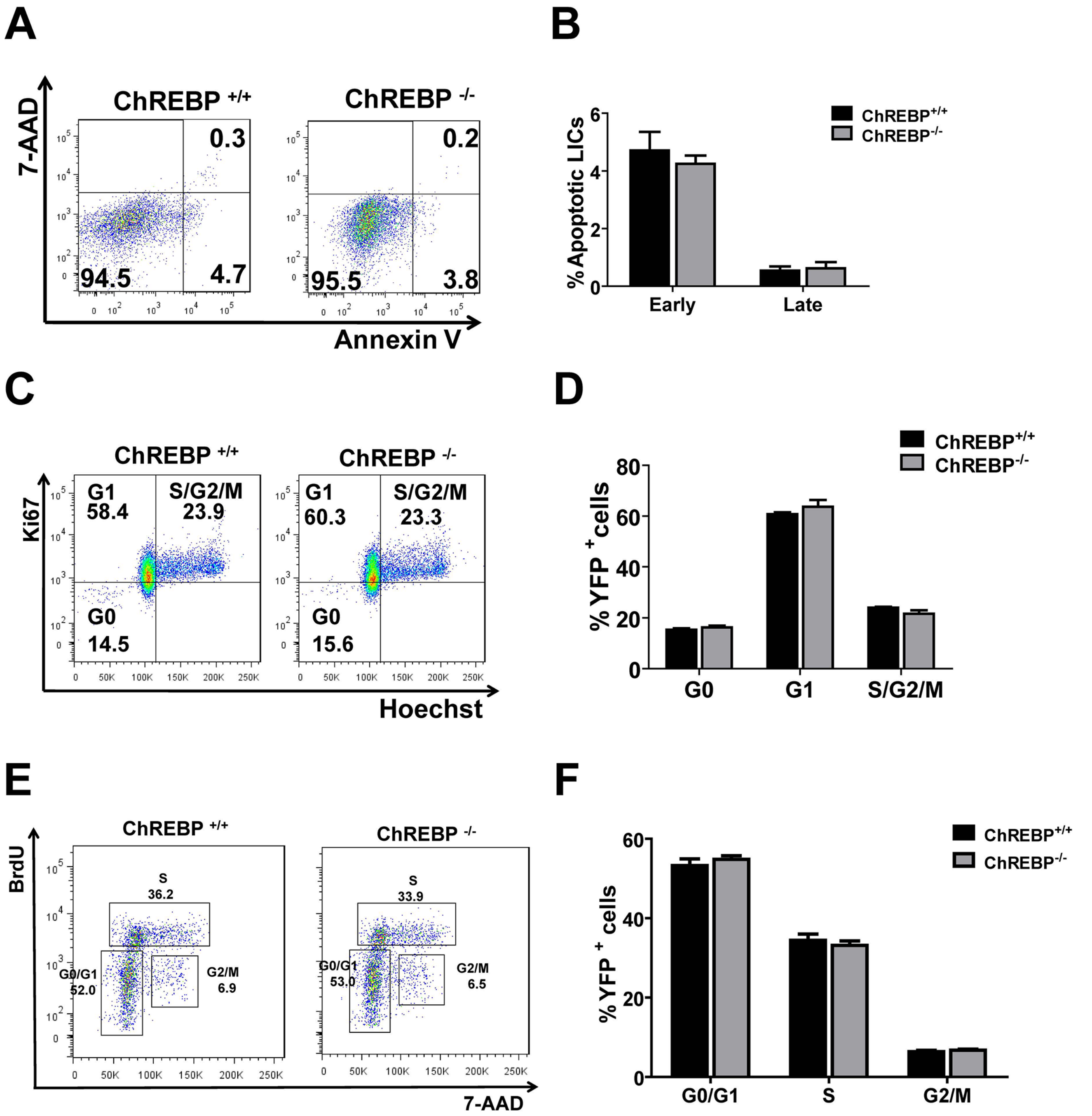

Supplementary Figure S2: No changes in apoptosis or the cell cycle of ChREBP-null LICs. (**A**) Representative flow cytometric analysis of cell apoptosis determined by Annexin V/7-AAD staining of WT or ChREBP-null LICs upon secondary transplantation. (**B**) Quantification of the frequencies of early and late apoptotic cells described in panel A (*n* = 3). (**C**) Representative flow cytometric analysis of the cell cycle measured by Ki-67/Hoechst 33342 staining of WT or ChREBP-null LICs upon secondary transplantation. (**D**) Quantification of the frequencies of cells in the G0, G1 and S/G2/M phases in WT or ChREBP-null LICs upon secondary transplantation (*n* = 3). (**E–F**) A BrdU incorporation assay was used to determine the cell cycle status of WT and ChREBP-null LICs.

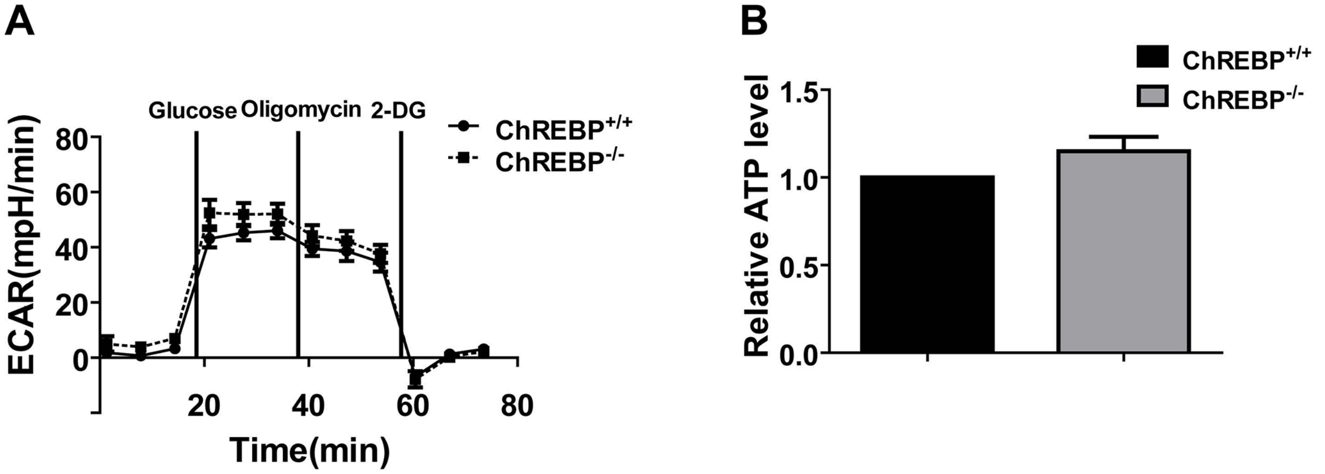

Supplementary Figure S3: Glycolytic flux analyses in WT and ChREBP-null AML cells. (**A**) Extracellular flux analysis revealed that ChREBP-null leukemia cells had similar ECARs compared with the WT controls (*n* = 3). (**B**) ChREBP-null leukemia cells had similar levels of ATP production compared with WT leukemia cells (*n* = 3).

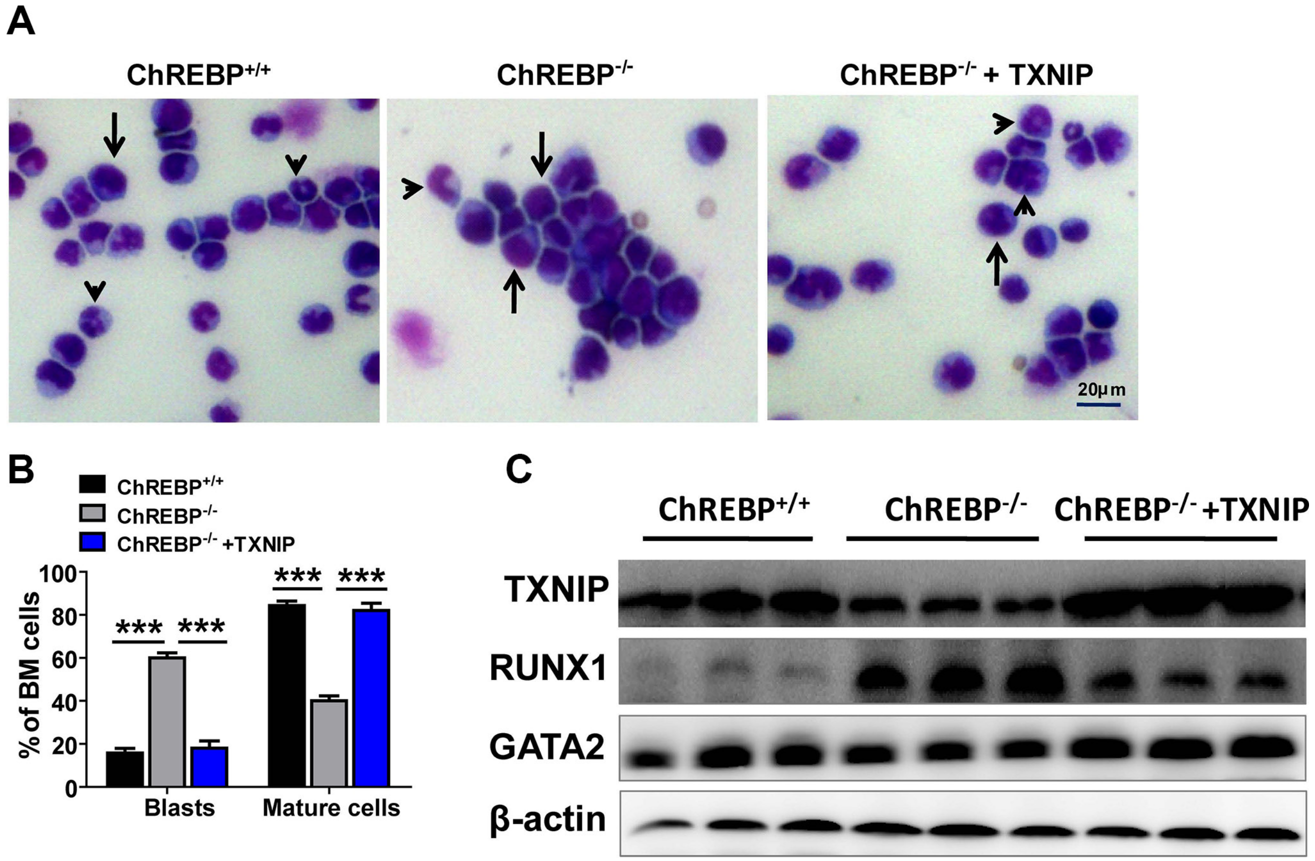

Supplementary Figure S4: Overexpression of TXNIP can rescue the loss of differentiation in ChREBP-null leukemia cells. (**A**) Representative images of Wright-Giemsa staining of WT, ChREBP-null, and TXNIP-overexpressing ChREBP-null BM leukemia cells in a rescue experiment. (**B**) Quantification of the blast cells (arrows) and mature cells (arrowheads) shown in panel A. A total of 15–30 cells were counted for each section and 8–10 sections were evaluated overall (*n* = 3). (**C**) TXNIP,RUNX1 and GATA2 levels were evaluated in WT, ChREBP-null and TXNIP-overexpressing ChREBP-null leukemia cells, respectively.

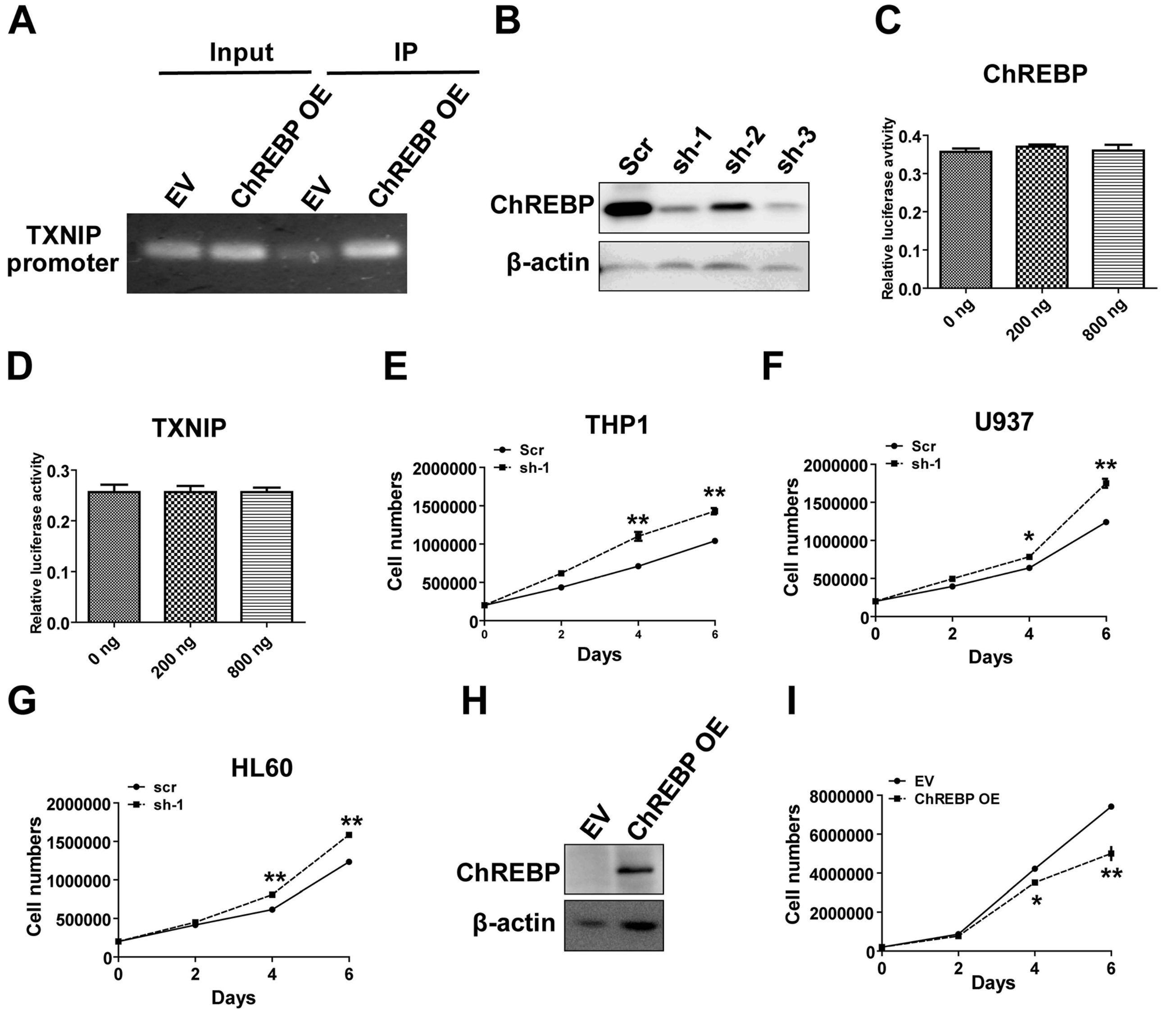

Supplementary Figure S5: ChREBP inhibits the proliferation of human AML cell lines. (**A**) ChIP assays were performed in ChREBP-overexpressing THP1 cells or control cells infected with empty vector (EV). PCR products showing the input control and the amplification of the ChREBP-binding sequence of TXNIP were measured by semi-quantitative PCR. (**B**) The knockdown efficiencies of ChREBP were evaluated by western blotting. (**C–D**) Luciferase activity was measured in 293T cells cotransfected with a GATA2 reporter and different amounts of ChREBP or TXNIP. (**E–G**) ChREBP was knocked down in human AML cell lines, including THP1 (E), U937 (F) and HL60 (G) cells, with the scrambled shRNA or shRNA-1(sh-1). Cell numbers were counted on the indicated days ($n = 3$). (**H–I**) The overexpression of ChREBP in THP1 cells was measured by immunoblotting and the cell numbers were calculated at the indicated time points. (*$p < 0.05$; **$p < 0.01$).
